# Drug-induced hyperpigmentation confounding clinical assessment and management for advanced chronic venous insufficiency

**DOI:** 10.1016/j.jvscit.2026.102327

**Published:** 2026-05-22

**Authors:** Mitali Doshi, Lara Sak, Juan Carlos Jimenez

**Affiliations:** Division of Vascular and Endovascular Surgery, Gonda Venous Center, David Geffen School of Medicine at UCLA, Los Angeles, CA

**Keywords:** Doxycycline, Hyperpigmentation, Minocycline, Stasis dermatitis, Venous insufficiency

## Abstract

Advanced chronic venous insufficiency (CVI) is commonly associated with lower extremity hyperpigmentation due to hemosiderin deposition. We present the case of a 56-year-old man with symptomatic bilateral CVI who underwent successful great saphenous vein radiofrequency ablation with resolution of pain and swelling but progressive ankle discoloration. Repeat duplex ultrasound examination confirmed durable vein closure without significant recurrent superficial disease. Dermatologic evaluation revealed chronic minocycline use, and biopsy demonstrated dermal pigment deposition positive for iron and Fontana-Masson stains, consistent with drug-induced hyperpigmentation. Discontinuation of minocycline led to partial improvement. This case highlights the importance of considering medication-related pigmentation in patients with skin discoloration and CVI.

## Abstract (es)

La insuficiencia venosa crónica (IVC) avanzada se asocia frecuentemente con hiperpigmentación de las extremidades inferiores debida al depósito de hemosiderina. Presentamos el caso de un hombre de 56 años con IVC bilateral sintomática, sometido exitosamente a ablación por radiofrecuencia de la vena safena magna, con resolución del dolor y del edema, pero con progresión de la hiperpigmentación a nivel del tobillo. Una ecografía dúplex de control confirmó el cierre venoso duradero, sin enfermedad superficial recurrente significativa. La evaluación dermatológica reveló uso crónico de minociclina, y la biopsia demostró depósito de pigmento dérmico positivo para las tinciones de hierro y de Fontana–Masson, consistente con hiperpigmentación inducida por fármacos. La suspensión de la minociclina condujo a una mejoría parcial. Este caso resalta la importancia de considerar también la pigmentación relacionada con medicamentos en pacientes con discromía cutánea e IVC.

Advanced chronic venous insufficiency (CVI) is often associated with skin discoloration and changes in texture and integrity. This can present as stasis dermatitis or lipodermatosclerosis and, in severe cases, can lead to venous ulceration. Venular hypertension causes erythrocytes to migrate across the microvascular network into the dermis, where they degrade hemoglobin into hemosiderin, which deposits in the dermis and causes hyperpigmentation.^1^ Many patients with advanced CVI seek comprehensive evaluation and intervention to improve associated lifestyle-limiting symptoms. As venous specialists, it is important to consider other possible concomitant pathological mechanisms for underlying skin changes in patients who present for vascular evaluation, because these can mask or confound the diagnosis of CVI. We present the case of a patient with bilateral venous insufficiency who continued to have progressive skin hyperpigmentation after bilateral radiofrequency ablation of the great saphenous veins (GSVs). Multidisciplinary management ultimately revealed the missed diagnosis of drug-induced hyperpigmentation. Consent was obtained from the patient for the publication of details and images for this case report.

## Case report

A 56-year-old man presented with long-standing bilateral lower-extremity discoloration, swelling, aching pain, and throbbing. His symptoms had been present for many years and were exacerbated by prolonged standing for several hours daily as part of his occupation in the furniture industry. He wore graduated (20-30 mm Hg) compression stockings daily without meaningful symptomatic relief. The year prior, he had a dermatologic biopsy of the left shin at an outside facility that demonstrated stasis dermatitis. Subsequently, he was referred for outpatient evaluation by a vascular surgeon.

**Fig 1:**
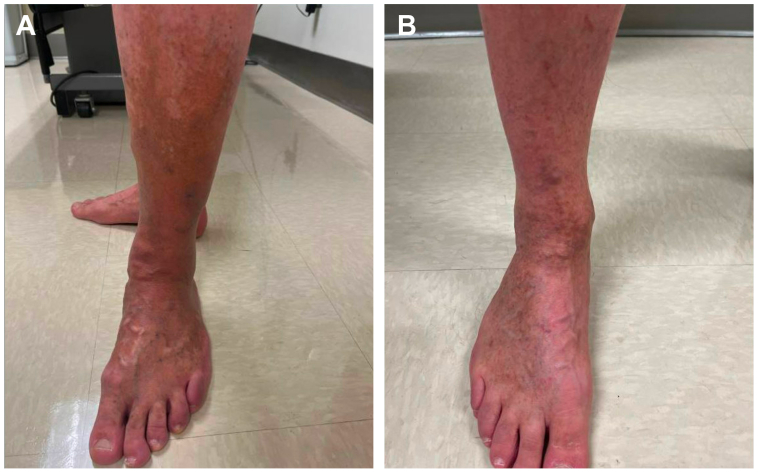
**(A, B)** Patient's bilateral leg skin discoloration and varicosities on initial evaluation.

On initial evaluation, his legs showed palpable varicosities and mild to moderate skin hyperpigmentation on the anterior aspects of the legs and ankles (Figs 1, *A*, *B*). Venous insufficiency duplex ultrasound evaluation demonstrated severe reflux (>4 seconds) of the bilateral GSVs and saphenofemoral junctions, with a maximal dilation of ≤8 mm in diameter. Bilateral below-knee GSV reflux (>3 seconds) was also present. He had no evidence of deep venous insufficiency or thrombosis. There was no perforator vein reflux noted. He subsequently underwent bilateral radiofrequency ablation of the GSVs, resulting in significant improvement in leg aching, swelling, and fatigue. These ablations were performed in a staged manner 1 month apart. Postprocedure duplex ultrasound examination 1 week after each procedure demonstrated successful closure of both GSVs with no evidence of ablation-related thrombus extension. No concomitant stab phlebectomies or sclerotherapy were performed.

**Fig 2:**
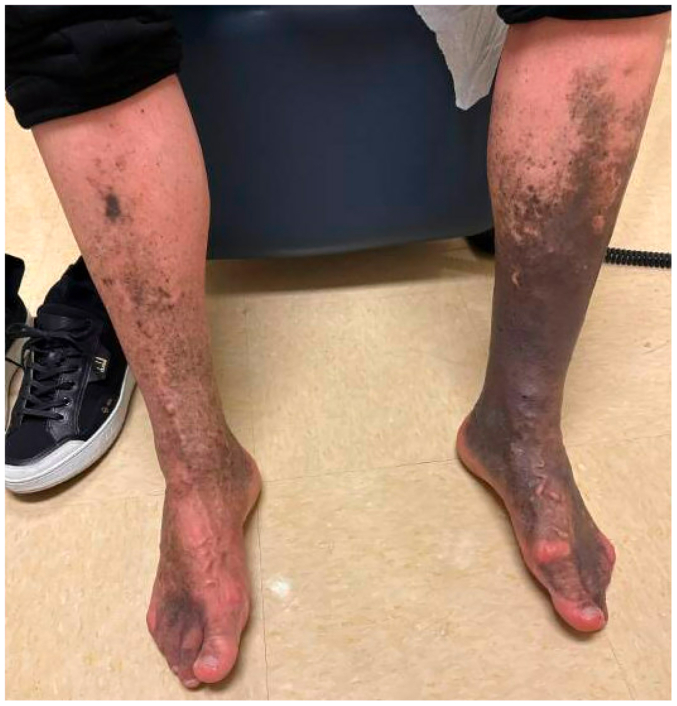
Patient's bilateral leg black discoloration 1 year after bilateral radiofrequency ablation of the great saphenous veins (GSVs).

One year later, the patient returned with complaints of progressive bilateral ankle and foot discoloration (Fig 2). He remained compliant with daily compression stockings use during the postoperative period. He did not have any evidence of hyperpigmentation to any other anatomical regions before or after his GSV ablations. Repeat duplex ultrasound examination demonstrated occluded bilateral thigh GSVs (from the groin to the knee), consistent with successful ablation. Additional findings included bilateral common femoral vein reflux, below-knee GSV reflux with small-caliber veins (approximately 2 mm), and superficial tributary vein reflux.

**Fig 3:**
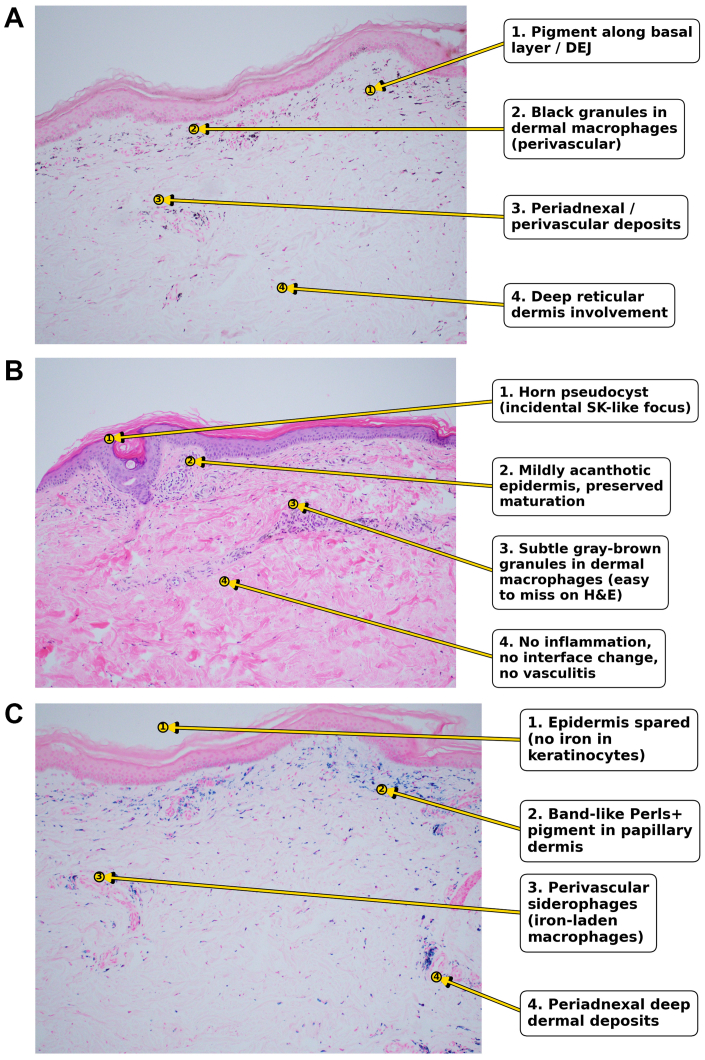
**(A)** Fontana-Masson stain demonstrating black granular pigment scattered throughout the papillary and reticular dermis. Pigment is also visible at the dermo-epidermal junction. **(B)** Routine hematoxylin and eosin (*H&E*) stain demonstrating gray-brown granular pigment within dermal macrophages. No significant inflammatory infiltrate is noted. **(C)** Perls’ Prussian blue stain demonstrating pigment concentrated within dermal macrophages and particular dense in the papillary and superficial reticular dermis. Pigment extends into the mid and deep reticular dermis. *DEJ*, Dermal-epidermal junction; *SK*, seborrheic keratosis.

The patient was referred for outpatient dermatologic evaluation. During this consultation, he reported long-term use of oral minocycline for acne for 10 years before his initial vascular surgery evaluation. He was also taking it at the time of his bilateral ablations. Dermatology performed a punch biopsy, which revealed findings compatible with drug-induced hyperpigmentation. Iron and Fontana-Masson stains were both positive within the dermal pigment (Figs 3, *A*-*C*). Neither hemosiderin nor malignancy were identified. The presence of pigment staining with both iron and Fontana-Masson was noted to be consistent with drug-induced hyperpigmentation, including that associated with minocycline use.

Minocycline was discontinued, and the patient was transitioned to doxycycline due to concern for medication-related pigmentation. After discontinuation of minocycline, the patient reported partial improvement in skin discoloration, although complete resolution did not occur. He continues to take doxycycline; the hyperpigmentation persists.

## Discussion

Minocycline is a tetracycline antimicrobial agent that works by binding to the bacterial ribosome and halting protein synthesis. It has anti-inflammatory and immunosuppressive properties, widely used for the treatment of many dermatologic conditions. Of all the tetracyclines, minocycline is most often associated with the adverse effect of pigmentation.^2^ The chemical compound is highly lipophilic and can readily penetrate lipid bilayers and distribute widely, and it can chelate metal ions to form insoluble complexes. This suggests a possible means by which insoluble minocycline complexed with iron may be deposited in diverse organs, including the skin, sclerae, bones of the oral cavity, oral mucosa, thyroid, heart valves, and bone.^3^^,^^4^^,^^5^^,^^6^^,^^7^^,^^8^ When the skin is involved, the pigmentation develops most frequently on the shins, ankles, and arms.^9^

Three distinct types of skin pigmentation occur: type 1 occurs on the face in areas of scarring and inflammation associated with acne, type 2 occurs on normal skin on the shins and forearms, and type 3 is a diffuse muddy brown discoloration in areas of sun exposure.^10^ Types 1 and 2 tend to resolve slowly over time, whereas type 3 persists indefinitely. Type 1 can develop after only a few weeks of therapy; in contrast, types 2 and 3 generally do not develop until patients have received a cumulative dose of minocycline exceeding 70 to 100 g.

Histopathology is characterized by deposition of brown/black, Fontana-Masson, and Perls’-positive granule deposits along elastic fibers in the papillary dermis and occurring within macrophages along vessels and eccrine units in dermis.^11^ In types 1 and 2 pigmentation, histopathology shows pigment granules in the dermis, within macrophages concentrated around vasculature, and around eccrine coils specifically in type 2. This pigment stains for both Fontana-Masson and Perls’ stains. For type 3 hyperpigmentation, histopathological findings are less specific, including increased melanin in basal keratinocytes and dermal melanophages, with no evidence of iron. Some case reports have also described histology showing sparing of the dermis and pigmentation, confined solely to the subcutaneous adipose tissue.^12^ Given the positive staining with Fontana-Masson and Perls’ stains in this patient, type 2 is more likely than type 3. However given the rapid development of worsened hyperpigmentation after thermal ablations, type 1 is also a possibility.

In addition to its effects on the skin, a published case report of a 44-year-old man with venous insufficiency resulting in a purpuric plaque on the right medial ankle demonstrates minocycline's effects on the vasculature.^13^ In this patient, ultrasound examination showed incompetence of the GSV and a large incompetent paratibial perforator vein immediately below the plaque. He also had a history of rosacea and was on minocycline for 5 months. He was treated with thermal ablation of the GSV and three sessions of sclerotherapy to occlude the associated tributaries. Two weeks post procedure, he had discolored tissue in a linear pattern on the skin from the medial thigh down to the medial calf, following the distribution of tributaries that were treated with foam sclerotherapy. A biopsy of the underlying vein from the anterior thigh revealed a staining pattern consistent with minocycline-induced pigmentation. This was the first report of venous tissue as a target of pigmentation. Minocycline may induce significant, potentially long term, pigmentation in predisposed patients undergoing sclerotherapy, with changes in their venous tissue, in addition to its effects on the skin.^13^ There have also been case reports of pigmentation of atherosclerotic plaques in the aorta and arteries in patients on chronic minocycline therapy.^14^ Laser therapy and chemical peel therapy may demonstrate some clinical benefit in these patients.

In patients evaluated by venous specialists for CVI, it is important to distinguish between postsclerotherapy pigmentation and drug-induced pigmentation (Table), including those caused by minocycline. Skin hyperpigmentation is a common complication after spider vein sclerotherapy.^15^ Usually, minocycline-induced hyperpigmentation persists beyond the 6-month to 2-year period of resolution expected in postsclerotherapy pigmentation.^1^ Hyperpigmentation caused by minocycline gradually fades when the medication is stopped, usually persisting for several months and sometimes years. Patients may consider holding minocycline during the perioperative period while undergoing thermal saphenous vein ablation to reduce inflammation associated with type 1 hyperpigmentation. However, there is no substantive literature or clinical guideline to strongly support this recommendation.

**Table:** Common drugs associated with hyperpigmentation

Drug	Color	Mechanism/histology	Reversibility
Minocycline	Blue-black, blue-gray, or muddy-brown (types I-III)	Iron-pigment complexes ± melanin in dermal macrophages; ↑ epidermal melanin in Type III	Slow; partial; Q-switched lasers help types I/II
Amiodarone	Slate-gray to violaceous	Lipofuscin-like lipid-drug complexes in dermal macrophages; phototoxic	Very slow (months to years); fades with discontinuation + photoprotection
Hydroxychloroquine/chloroquine	Blue-gray to black	Melanin + hemosiderin deposition; often follows ecchymoses	Slow improvement after discontinuation
Bleomycin	Brown (flagellate linear streaks)	↑ melanogenesis; possible postinflammatory mechanism	Usually fades over months
Busulfan and other cytotoxics (cyclophosphamide, 5-FU, doxorubicin)	Diffuse brown	↑ epidermal melanin; direct melanocyte stimulation	Often persistent
Chlorpromazine/phenothiazines	Slate-gray to violet	Drug-melanin complexes in dermis; phototoxic	Slow, often incomplete
Zidovudine (AZT)	Brown (skin, nails, mucosa)	↑ melanin in basal keratinocytes	Reversible with discontinuation
Estrogens/oral contraceptive pills	Tan-brown (melasma)	Hormonal stimulation of melanogenesis	Often persistent; responds to triple combination + photoprotection
Heavy metals — silver (argyria), gold (chrysiasis)	Slate-gray (silver), blue-gray (gold)	Metallic particle deposition in dermis	Permanent

↑, Increased; *5-FU*, 5-fluoracil.

## Conclusions

This case illustrates a rare and instructive presentation of CVI with progressive lower extremity discoloration after successful bilateral GSV ablation, in which the predominant contributor to worsening pigmentation was ultimately identified as drug-induced hyperpigmentation rather than recurrent venous pathology. To our knowledge, it is the first such publication in this particular context. It underscores the importance of obtaining a comprehensive medication history during initial and follow-up venous evaluations and highlights how changes in venous stasis may obscure alternative etiologies of lower extremity hyperpigmentation. In this patient, a multidisciplinary approach was essential in arriving at the correct diagnosis and guiding appropriate management.

## Funding

None.

## Disclosures

J.C.J. is on the advisory board for Boston Scientific (100% of earnings donated to charitable foundations).
